# Cohort profile: the Entebbe Mother and Baby Study (EMaBS)

**DOI:** 10.1136/bmjopen-2026-118325

**Published:** 2026-08-26

**Authors:** Emily L Webb, Bridgious Walusimbi, Ronald Komata, Alison M Elliott, The EMaBS Study Team

**Affiliations:** 1 MRC/UVRI and LSHTM Uganda Research Unit, Entebbe, Uganda; 2 Infectious Disease Epidemiology and International Health Department, London School of Hygiene & Tropical Medicine, London, England, UK; 3 Infection Biology Department, London School of Hygiene & Tropical Medicine, London, England, UK; 4 Clinical Research Department, London School of Hygiene & Tropical Medicine, London, England, UK

**Keywords:** Immunity, INFECTIOUS DISEASES, Africa South of the Sahara, Longitudinal studies, EPIDEMIOLOGIC STUDIES

## Abstract

**Purpose:**

The Entebbe Mother and Baby Study (EMaBS) was established in 2001 to test the hypothesis that treating helminth infections during pregnancy and early childhood could improve children’s responses to Bacillus Calmette-Guérin (BCG) and other vaccines given in infancy and influence immune responses to other infectious pathogens. Follow-up was subsequently extended to address further research questions and continue to the present day.

**Participants:**

Two thousand five hundred and seven pregnant women were recruited when attending antenatal services at Entebbe General Hospital, Uganda; 2345 resulting live-born children were enrolled into the EMaBS birth cohort.

**Findings to date:**

Initial results from EMaBS showed that treating helminths in pregnancy and early childhood was safe and that treatment with albendazole reduced anaemia in mothers with heavy hookworm infections. Maternal anthelminthic treatment had small effects on infant response to tetanus immunisation, but no effect, either beneficial or detrimental on the occurrence of infectious diseases in childhood. However, treatment of helminths during pregnancy resulted in increased rates of eczema in early childhood, although this was not sustained to nine years. Subsequent work in early adolescence found that postnatal weight gain was important in the developmental programming of blood pressure in this population and current and early-life malaria modified blood pressure and lipid levels. We also showed that variation in host genes significantly shapes antibody responses to multiple childhood vaccines, highlighting genetics as a key determinant of vaccine performance.

**Future plans:**

Cohort ‘children’ are currently aged 19–22 years, and future plans focus on investigating longer term effects of early-life and childhood exposures. A new round of data collection is ongoing with the aim of determining the impact of early-life exposures on adult non-communicable disease risk. Work determining whether frequent childhood infections lead to specific epigenetic changes implicated in later disease development is also underway.

**Registration:**

The EMaBS began as a randomised controlled trial (ISRCTN32849447). Two further randomised controlled trials have been nested within the cohort: TB042 (NCT03681860) and POPVAC C (ISRCTN10482904).

**STRENGTHS AND LIMITATIONS OF THIS STUDY**

- A key strength of the cohort is the collection of detailed prenatal and childhood data on nutrition, growth, infectious and sociodemographic characteristics, supplemented by genetic data and stored samples.
- Strong community and stakeholder engagement, and provision of free medical care to cohort mothers and children have been key in ensuring sustained participation from cohort members, continuing into adulthood.
- Entebbe Mother and Baby Study (EMaBS) has made a sustained contribution to research capacity strengthening in Uganda, including supporting prevention of mother-to-child HIV transmission programme implementation at Entebbe Hospital, training 150 local community field workers, providing continuous professional development for research and clinical staff and enabling university training through 75 internships and 12 PhD projects, creating a skilled workforce and a lasting research infrastructure.
- Selection bias and differential attrition may have implications for the representativeness of cohort members still under active follow-up.
- A potential limitation in the use of EMaBS data for secondary analyses includes the possibility of residual confounding from unmeasured factors, particularly for new research questions that were not envisaged when the cohort was initiated.

## Introduction

 The Entebbe Mother and Baby Study (EMaBS) began in 2001 as a collaboration between Entebbe Hospital, Entebbe Municipality, the London School of Hygiene and Tropical Medicine (LSHTM) and the Medical Research Council (MRC)/Uganda Virus Research Institute (UVRI) Uganda Research Unit (MUL) on AIDS (now, since 2018, the MRC/UVRI and LSHTM MUL). Our concern at the time was to understand why Bacillus Calmette-Guérin (BCG) - still the only licenced vaccine for tuberculosis - is less effective in countries close to the equator than in temperate countries such as the United Kingdom, and in rural compared with urban settings in those countries.^1 2^ Evidence had emerged that parasitic worm infections generate a profile of immune responses (Th2), which oppose the responses likely to protect against tuberculosis (Th1 responses).^3^ BCG is commonly given at birth, and work in Kenya had shown that exposure in utero to worms in the mother could bias BCG responses in the offspring in a Th2 direction.^4^ We thus designed EMaBS to test whether treating worms during pregnancy could improve the baby’s response to BCG and other infant vaccines. To address this aim, we recruited 2507 pregnant women into a randomised controlled trial of anthelminthic treatment and followed up the resulting offspring, thus initiating a birth cohort with a wealth of data on early-life infection exposure and treatment.

In 2001, as we prepared to start our project, Uganda was just beginning to roll out a programme for prevention of mother-to-child HIV transmission (PMTCT) using single-dose nevirapine, but this was not yet in place at Entebbe Hospital. Hospital staff and the research team worked together closely to implement PMTCT and a strong antenatal care programme (providing syphilis testing and treatment, haematinics and presumptive treatment of malaria with sulfadoxine/pyrimethamine) and to assess uptake of HIV testing and other interventions among antenatal women and their partners.^5 6^ As well, the microbiology laboratory at MUL undertook preparatory studies on the assessment of worm infections during pregnancy.^7^

At first, our trial was designed to test the effects of albendazole (which treats soil-transmitted helminths including hookworm, *Trichuris*, *Ascaris* and *Strongyloides*) compared with placebo in pregnancy.^8^ But soon after we had started our study, the WHO reviewed the potential benefits of treating schistosomiasis with praziquantel in pregnancy and called for trials of its safety and benefits.^9^ Thus, we paused the trial after enrolling 103 participants. This small ‘pilot’ group provided some important initial insights, which guided future work.^8 10^ We updated our trial design to a 2×2 factorial design comparing albendazole versus placebo and praziquantel versus placebo during pregnancy. We also included a third randomisation of albendazole versus placebo given quarterly in the offspring, from 15 months to 5 years, since the benefits of deworming in very young children were also uncertain.^11^

**Figure 1:**
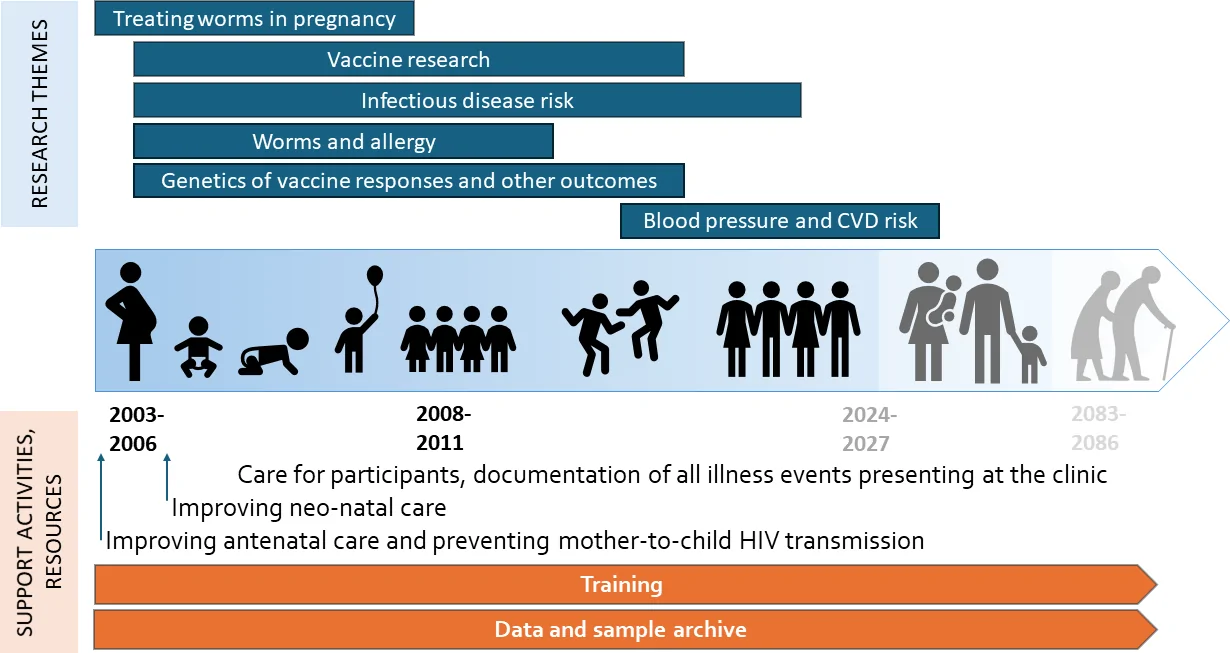
Entebbe Mother and Baby Study, 2001–2025. Showing the timeline of the Entebbe Mother and Baby Study and summarising the main research themes, supporting activities and resources. CVD: cardiovascular disease; HIV: human immunodeficiency virus.

Following the conclusion of the original trial interventions when the children reached 5 years of age, the EMaBS participants have continued to be followed up as a birth cohort, evolving over time into a multidimensional cohort focusing on concepts of immune development, infection susceptibility, nutritional status and, more recently, the developmental origins of cardiovascular diseases. Figure 1 summarises the cohort timelines and main research themes. Between 2024 and 2027, cohort ‘children’ are reaching 21 years of age and new work is in progress to determine the impact of early-life exposures on adult non-communicable disease risk through pathways such as infection-driven epigenetic changes. Here, we describe characteristics of the EMaBS cohort, its major scientific contributions to date, and our present and future research priorities. We also describe how EMaBS offers opportunities for the wider research community to address pertinent global health questions, including how early life exposures influence lifelong disease risk.

## Cohort description

### Setting

**Figure 2:**
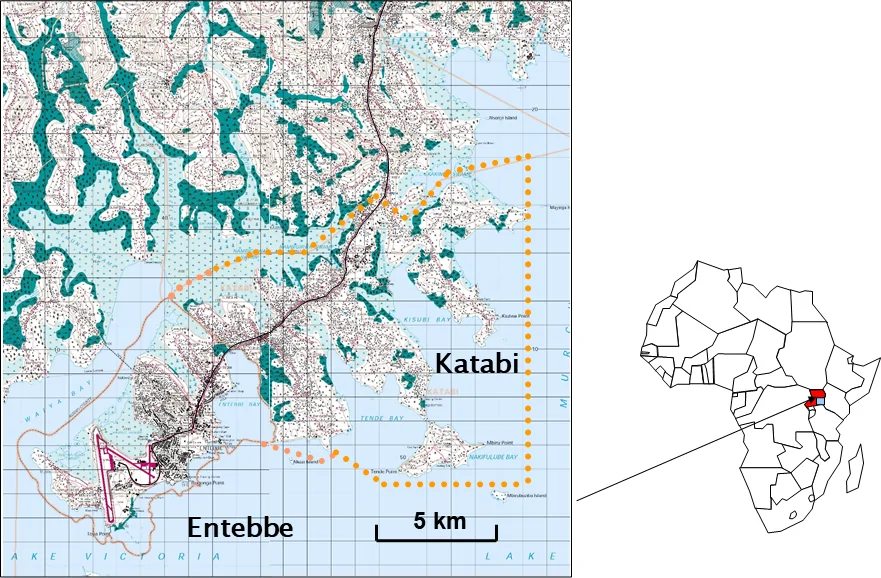
Location of the Entebbe Mother and Baby Study.

The EMaBS is based in Entebbe Municipality and surrounding areas of Katabi subcounty, Central Uganda. This is a diverse setting comprising urban, peri-urban and rural communities and fishing communities on the shores of Lake Victoria (figure 2).

### Recruitment and eligibility

Pregnant women were recruited between 7 April 2003 and 1 November 2005 when attending antenatal services at Entebbe General Hospital. Approximately 60%–80% of pregnant women in this community received antenatal services at Entebbe General Hospital during the recruitment period.^12^

**Figure 3:**
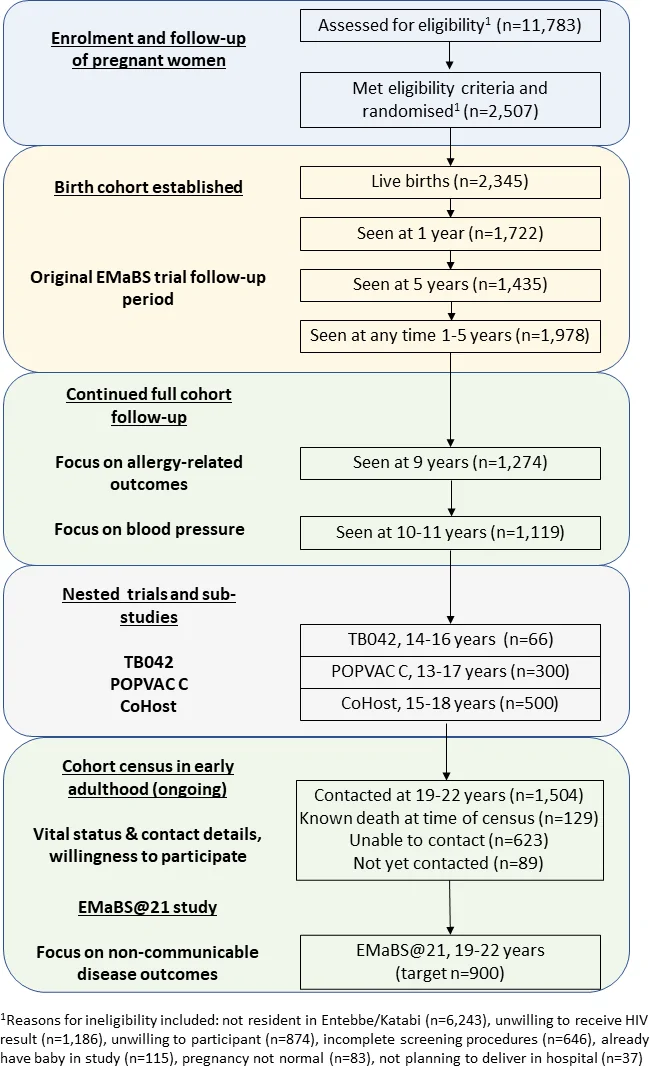
Flow diagram of Entebbe Mother and Baby Study participants. Flow diagram indicating the number of participants seen at key stages of the cohort. EMaBS, Entebbe Mother and Baby Study.

Women were eligible for recruitment if they were well on the day of recruitment, resident in the study area, planning to deliver at the hospital, willing to know their HIV status and prepared to participate in the study. They were excluded if they had haemoglobin below 8 g/dL, clinically apparent severe liver disease, history of diarrhoea with blood in stool, abnormal pregnancy, history of adverse reaction to antihelminthics or had already been enrolled during an earlier pregnancy. A total of 2507 women were enrolled and randomised to receive either albendazole (400 mg) or matching placebo, and praziquantel (40 mg/kg) or matching placebo, in a 2×2 factorial design. They were then followed to delivery and 2345 resulting liveborn children, born between 17 April 2003 and 19 April 2006, were enrolled into the EMaBS birth cohort. No further eligibility criteria were applied to the children. At age 15 months, children were randomised to receive either albendazole (200 mg in form of syrup, from age 15 to 21 months; 400 mg in form of chewable tablet, from age 2 to 5 years) or placebo quarterly. Written informed consent was obtained two times: from the mother during pregnancy, and from the mother or caregiver when the child reached age 1 year, for taking part in the childhood intervention. Figure 3 outlines screening and enrolment and the number of participants seen at key points during cohort follow-up. To date, 129 EMaBS participants are confirmed to have died, the majority (90, 70%) in infancy. There have been no formal withdrawals. An ongoing census has confirmed updated contact details and willingness to continue taking part in cohort activities for 1504 participants. A total of 623 participants (28% of those not known to have died) could not be contacted, and for the remaining 89 of the original 2345 live-born children, attempts to contact are ongoing.

### Data collection and follow-up procedures

An overview of the main data and sample collection activities to date is provided in table 1.

**Table 1:** Summary of data collected to date and stored samples in the Entebbe Mother and Baby Study

Time point	Number of participants	Key data and stored samples
**Core activities**		
Enrolment in pregnancy (maternal data)	n=2507	**Data**: sociodemographic, parental characteristics, full blood count, anthropometry, infections, blood pressure, immunology **Samples**: plasma, serum, cell pellet
Delivery (maternal and child data)*	n=2389	**Data**: birth weight, delivery details, congenital anomalies, infections (maternal), FBC, immunology **Samples**: plasma, serum, cell pellet (mother and cord blood)
Infancy	n=2345 live births	**Data**: anthropometry (several time points), immunisations, immune response to immunisations; HIV status (vertically exposed infants)
Continuously, from birth	All	**Data**: illnesses—participants brought to study clinic when unwell
Annually, from 1 year	All	**Data**: anthropometry, infections, recalled illnesses, full blood count (≤5, 9, 10/11 years), immunology, blood pressure (≥10 years) **Samples**: plasma, serum, cell pellet, stool
Not applicable	n=1391	**Data**: whole genome data on 2.2 million variants
**Additional data collected from all available cohort participants at specific annual visits**		
9 years	n=1274	**Data**: allergy-related diseases and atopy (skin prick tests and IgE)
10-11 years	n=1119	**Data**: blood pressure, lipid levels (both n=1119), body composition (n=177)
**Additional data collected through nested studies**		
1–5 years^1^	n=1391	**Data:** micronutrients and inflammation including measures of iron status, vitamin D levels and C reactive protein
5 years	n=870	**Data**: cognitive function (n=870), home environment (n=163)
14–15 years	TB042 trial, n=66	**Data**: sociodemographic, anthropometry, immunisation, response to immunisation **Samples**: plasma, serum, cell pellet, PBMCs, PAXgene, supernatant, stool
15–17 years	POPVAC C trial, n=300	**Data**: sociodemographic, infections, treatment, immunisations, response to immunisations **Samples**: plasma, cell pellet, tempus, PBMCs, supernatant, stool, urine (multiple time points)
16–18 years	CoHost study, n=500	**Data**: sociodemographic/household characteristics, SARS-CoV-2 serology, immunisation and response, COVID-19 disease **Samples:** plasma, PAXgene, cell pellet, PBMCs, stool (multiple time points)
**Census (April 2023–June 2026)**		
18-22 years	n=1504	**Data:** up-to-date contact details, willingness to take part in future data collection

* Information was captured as soon as possible after delivery, regardless of delivery location. From a single time point in this age range. FBC, full blood count. PBMCs, Peripheral blood mononuclear cells

POPVAC, POPulation differences in VACcine responses.

All assessments and data collection activities were conducted at the EMaBS clinic at Entebbe Hospital, with most laboratory results generated either at the clinic or at the main MRC/UVRI and LSHTM Uganda Research Unit laboratories; exceptions include the genetic data, which were generated at the Wellcome Sanger Institute, and the micronutrient data which were generated at the University of Oxford. At all visits, questionnaires were delivered to study participants and (for children) their caregivers by trained clinicians, nurses and field workers. Physical examinations were conducted by clinicians.

Information on a broad array of sociodemographic characteristics, history of illness, anthropometry and infections was collected from women at enrolment. At delivery, data on the location and type of delivery, any complications or congenital anomalies, and birth weight were recorded. Children were followed up for routine immunisations and quarterly for provision of trial interventions. Once a year, close to their birthdays, children were invited to attend an ‘annual visit’ at which information on anthropometry and history of any illnesses in the past year was collected, and blood and stool samples tested for asymptomatic malaria and helminth infections, respectively. Children were treated appropriately if infection was detected. Concurrently, caregivers were invited to bring children to the clinic for treatment when sick, at no cost. Laboratory data generated from samples collected at annual visits include immune responses to vaccinations at 1 and 5 years and serial serostatus information for common viruses.

In 2012, an amendment to the protocol was approved supporting genetic studies within EMaBS to address outcomes related to vaccine responses, infectious diseases and inflammation. We explained these plans to participants, and they were supportive, almost all giving consent. The development of this genetic resource and partnerships with other African cohorts has created further important opportunities, as detailed below.

Following completion of the original trial follow-up period at age 5 years, children continued to be followed up annually and invited to attend the clinic if unwell. Additional focused activities were done at 9 and 10–11 years to assess allergy-related diseases and blood pressure and lipid levels, respectively, among all available cohort participants. Between ages 14 and 18 years, subsets of participants took part in nested studies: (1) the TB042 trial, which compared the safety and immunogenicity of regimen ChAdOx1 85A–MVA85A with BCG revaccination^12^; (2) the POPulation differences in VACcine responses (POPVAC) C trial, which examined the effect of BCG revaccination on responses to other unrelated vaccines^13 14^; (3) the CoHost study which examined the effect of early-life and concurrent exposures on response to SARS-CoV-2.^15^ As described in the future plans section, new data collection and generation is ongoing and planned, focussing on non-communicable disease outcomes and epigenetics. Selected characteristics of enrolled women and the resulting birth cohort are indicated in table 2.

**Table 2:** Selected characteristics of Entebbe Mother and Baby Study participants at birth and during early childhood, and of their mothers at enrolment in pregnancy

Characteristic		N (%) unless otherwise indicated
**Maternal characteristics at enrolment (n=2507 participants)**		
Age in years	Mean (SD)	23.6 (5.3)
Education (highest completed)*	None	97 (4)
	Primary	1263 (50)
	Secondary	934 (37)
	Tertiary	209 (8)
Marital status*	Single	336 (13)
	Married	2099 (84)
	Widowed/divorced/separated	71 (3)
Gravidity	*	695 (28)
	2–4	1412 (56)
	5+	400 (16)
Hookworm infection*	No	1386 (55)
	Yes	1112 (45)
*Schistosoma mansoni* infection*	No	2040 (82)
	Yes	458 (18)
*Strongyloides stercoralis* infection	No	2179 (88)
	Yes	306 (12)
*Trichuris trichiura* infection	No	2272 (91)
	Yes	226 (9)
*Ascaris lumbricoides* infection	No	2440 (98)
	Yes	58 (2)
*Mansonella perstans* infection*	No	1968 (79)
	Yes	531 (21)
Malaria parasitaemia*	No	2191 (89)
	Yes	268 (11)
HIV status	Negative	2207 (88)
	Positive	300 (12)
**Child characteristics (n=2345 live births)**		
Sex*	Male	1201 (52)
	Female	1130 (48)
Birth weight*	Mean (SD)	3.2 (0.5)
Mode of delivery*	Normal	2051 (91)
	Caesarean/assisted	200 (9)
Perinatal HIV status	Unexposed	2065 (88)
	Exposed, uninfected	167 (7)
	Exposed, infected	54 (2)
	Exposed, unknown status	59 (3)
Number of clinical malaria events diagnosed at EMaBS clinic, 0–10 year	Median (IQR)	1 (0, 2)
Asymptomatic malaria, any annual visit, 1–5 years*	No	1765 (84)
	Yes	334 (16)
*Trichuris trichiura* infection at any annual visit, 1–5 years*	No	1916 (91)
	Yes	179 (9)
*Ascaris lumbricoides* infection at any annual visit, 1–5 years*	No	2005 (96)
	Yes	90 (4)
*Schistosoma mansoni* infection at any annual visit, 1–5 years*	No	2036 (97)
	Yes	59 (3)
Hookworm infection at any annual visit, 1–5 years*	No	2047 (98)
	Yes	48 (2)

* Missing data for maternal education (n=4), marital status (n=1), hookworm (n=9), *S. mansoni* (n=9), *S. stercoralis* (n=22), *T. trichiura* (n=9), *A. lumbricoides* (n=9), *M. perstans* (n=8), malaria parasitaemia (n=48), child sex (n=14), birth weight (n=444), mode of delivery (n=94), childhood asymptomatic malaria (n=246), childhood helminth infection (n=250).

EMaBS, Entebbe Mother and Baby Study.

### Patient and public involvement

Involvement of the participants and the broader community has been fundamental to study preparation, enrolment and ongoing follow-up. During the planning stages of the study, meetings were held with community leaders to explain the planned research questions and obtain input into study conduct. Each village in the study area has an elected committee, and each of these committees appointed volunteer assistants (‘community field workers’), members of the public who have played a key role in the study. Following initial training and certification, community field workers followed up EMaBS children two times a month to age 5 years and attended monthly update meetings. Each received a bicycle and small allowance to support these activities. They continue to provide valuable support to EMaBS with follow-up and engagement activities, contributing their insights gained from these activities to inform the design and implementation of the research. More recently, the MRC/UVRI and LSHTM Uganda Research Unit has established a community advisory board who have also been engaged in meetings discussing new planned work in the cohort and recent findings. Throughout the course of the study, participants and their parents and caregivers have taken part in engagement meetings; these have predominantly involved large-scale dissemination days at which results have been shared and up-coming proposed work has been discussed. Our project website also provides information and updates on the latest cohort activities.^16^

## Findings to date

### Safety of treating worms during pregnancy

Among the mothers, infections were common (table 2); we fine-mapped the distribution of worm and malaria infection prevalence in Entebbe and the surrounding areas,^17^ informing understanding of differences in exposures between more rural and more urban elements of the catchment area and documented risk factors for these infections in pregnancy.^18^

The risks and benefits of using benzimidazoles, such as albendazole, and praziquantel during pregnancy were still a matter of debate as we embarked on our trial. We studied birth outcomes carefully^19^ and found no evidence that these interventions were harmful.^20^ We subsequently contributed our data to support the case for using these treatments for pregnant women in endemic settings, particularly to avoid their exclusion during mass drug administration.^21 22^

One of the main reasons advanced for treating pregnant women for worms is to reduce their risk of anaemia. In our cohort, hookworm was the most common worm infection in pregnancy (44%) and anaemia was associated with hookworm intensity^23^; there was some evidence that treatment with albendazole improved haemoglobin levels in mothers with heavy hookworm infection.^24^ However, maternal anaemia was associated more strongly with malaria and HIV than with any worm infection.^23^

### Vaccine responses and infectious diseases in infancy and early childhood

We hoped that treating maternal worm infections would result in enhanced immune responses, among their babies, to the vaccines given by the Expanded Programme on Immunisation, and increase the babies’ resistance to common infections such as malaria, diarrhoea and pneumonia. We found that this was not the case,^24–26^ and indeed, in secondary analyses, there was little to suggest an association between maternal worm infections and infant vaccine responses.^27^ However, childhood malaria and HIV infection, and for some vaccines anthropometric measures of poor nutrition, were associated with reduced responses to BCG, tetanus and measles antigens, suggesting a more important impact of exposure to direct comorbidities.^27–29^ These analyses also highlighted that maternal tetanus immunisation during pregnancy enhanced infant responses to tetanus immunisation, and that BCG scarring and responses differed according to the BCG vaccine strain that an infant received.^27 29 30^

With regard to infectious disease risk, we examined tuberculosis infection status at age 5 years using a T-SPOT.TB assay.^29 31^ There were no associations with prenatal exposure. There was weak evidence of an association with Th2-biased responses to tuberculosis antigen at age 1 year, but the principal risk factors were urban living and contact with a tuberculosis patient.^29^ A further observational analysis indicated increased risk of malaria in the first 5 years of life in the children of mothers with worms during pregnancy, with the strongest effect in children of mothers with hookworm. These findings were robust after taking into account potential confounding, particularly related to location of residence, and seemed stronger when the mother was coinfected with worms and malaria.^32^ Maternal anthelminthic intervention showed no effect on malaria incidence, however, suggesting that if the effects of prenatal worm-malaria coexposure were causal they could not be reversed by subsequent treatment.^24 25^ The newly implemented PMTCT programme reduced vertical HIV transmission to 18%. Hookworm and *Trichuris* infections were associated with increased HIV load in pregnancy and albendazole reduced viral load 6 weeks after treatment, but this effect diminished by the time of delivery.^33^ Children born to mothers living with HIV but who were themselves uninfected were at increased risk of being underweight, but no other adverse effects of HIV exposure were seen.^34^

EMaBS also made a particular contribution to studies on Kaposi Sarcoma Herpesvirus (KSHV) in Uganda. KSHV is highly prevalent in the region, although more so in rural than urban settings.^35^ We discovered positive associations of malaria and helminths with KSHV titres in EMaBS mothers^36 37^ and between malaria and KSHV titres in children.^37 38^ We showed, in EMaBS children, that younger age of KSHV infection is associated with higher KSHV antibody titres (suggestive of more active latent infection) and that KSHV shedding in mothers and children is associated with malaria exposure.

Most of the analyses described so far explored effects of worms and malaria in pregnancy and early childhood on unrelated infections. Since EMaBS was the first study to implement praziquantel treatment for schistosomiasis during pregnancy and used a randomised design such that half the *S. mansoni*-infected mothers were treated during pregnancy, and half after delivery, we investigated effects on cellular and humoral responses to schistosome antigens in the mothers. Treatment of the mothers boosted these antigen-specific responses, but the boosts were lower when treatment was given during pregnancy than after delivery.^39 40^ We investigated effects of maternal treatment on the children’s responses to schistosome antigens and infection,^41 42^ but by age 5 years, only 2% of children were infected and no effect of maternal treatment on infection status was seen. Children of treated mothers had higher schistosome-specific IL-10 responses, suggesting a possible effect on regulation of immune responses and possibly the risk of later pathology.^42^

### Genetics

Collaborating with other African cohorts through the VaccGene consortium coordinated by colleagues at the Wellcome Sanger Institute and University of Oxford, we contributed to work demonstrating that genetic factors influence the response to pertussis, diphtheria and hepatitis B vaccines, explaining up to 10% of antibody response variance.^43^ For pertussis, we found evidence that *HLA-DRB1* gene expression is a factor in vaccine-induced protection against this important infectious disease.^43^

### Vaccine responses in adolescents

During the early years of EMaBS, increasing evidence emerged that regional differences in BCG efficacy were more important when the vaccine was given to adolescents than when it was given at birth, suggesting effects of cumulative, direct environmental exposures, rather than prenatal exposures.^44^ This is of concern because an effective vaccine against adult, pulmonary (infectious) disease is needed to control the global tuberculosis pandemic. In addition, there was increasing evidence that several vaccines of different types showed similar variations in efficacy and immunogenicity.^45–49^ We, therefore, undertook a programme of work on POPVAC in three settings in Uganda, with adolescents in the EMaBS cohort acting as relatively urban participants, for comparison with rural schistosome-endemic and malaria-endemic settings.^14 50^ We found striking differences between the three settings, including in responses to BCG, yellow fever, live, oral typhoid vaccine and tetanus vaccines. In mediation analyses, there was little evidence to support the hypothesis that malaria or schistosomiasis were primary drivers of these differences, but some evidence that differences in prior exposure to the vaccine-target, or related, cross-reactive infections play a role.^13^ More detailed studies involving EMaBS adolescents and those from the schistosome-endemic islands showed evidence that schistosomiasis has some adverse effect on responses to BCG vaccination and oral typhoid vaccine^51^; also that schistosomiasis causes both intestinal inflammation and increased intestinal permeability, systemic effects on the immune system, which may influence vaccine responses.^52^ Going forward we are using POPVAC samples to understand these results better, and hope to find ways to improve vaccine responses (and efficacy) in settings, and among individuals, where they are impaired.

One solution to the relatively poor efficacy of BCG given in adolescence against adult, pulmonary tuberculosis might be to develop a different type of vaccine, less susceptible to environmental effects. We, therefore, undertook a further trial within EMaBS, comparing the immune response to BCG revaccination among adolescents with the immune response to a new, viral-vectored booster regime: ChAd85A followed by MVA85A. We found that the new regimen induced better antibody and cellular responses to the vaccine antigen (85A) than the BCG revaccination, supporting plans to further develop this type of approach for boosting protection against tuberculosis among young adults.^12^

### Effects of worms and their treatment on allergy-related disease outcomes

While our original intention was to investigate effects of worms and their treatment on vaccine responses and infectious disease outcomes, an early indication of possible effects on allergy-related outcomes was given by observations in the ‘pilot’ group where mothers were randomised only to albendazole or placebo. We noticed unexpectedly high rates of infantile eczema, and that maternal helminth infection appeared to be protective, with some evidence of an adverse effect of albendazole treatment.^10^ We amended the main trial protocol to test this further and worked with EMaBS mothers to establish a suitable panel of antigens for skin prick testing, to determine atopic sensitisation, in this setting.^53^ In the main trial, we obtained strong evidence that treatment with albendazole or praziquantel during pregnancy increased the risk of eczema in the children.^54^ This was hailed as the first conclusive evidence that an intervention during pregnancy can alter allergy-related disease risk in the offspring—although, in this case, in an adverse direction. Curiously, the effect of albendazole was not restricted to children of mothers infected with the worm species that it treats (whereas the effect of praziquantel was restricted to children of schistosome-infected women) raising uncertainty about the mechanism of albendazole treatment effects. However, evidence of a protective effect of maternal helminths was strengthened by further analysis, showing that maternal hookworm modified the adverse effects of other risk factors, such as family history and allergen sensitisation, as well as the adverse effects of albendazole treatment.^55^

Since infantile eczema can be linked to later risk of asthma, these findings raised concerns that treating worms during pregnancy might increase later asthma risk in the children. We investigated this, documenting eczema and asthma events prospectively, and collecting detailed data on atopic sensitisation and symptoms of allergy-related diseases when children were 9 years old. However, we found that, although atopic sensitisation increased with age, allergy-related disease prevalence declined (with eczema, rhinitis and wheeze each 5% or less at 9 years).^56^ Latent class analysis identified groups of participants characterised by higher rates of malaria, diarrhoea and lower respiratory tract infection (LRTI) in infancy, and of helminths, malaria and LRTI from birth to 5 years, and that children in these groups had lower risk of allergy-related disease at age 9 years.^57^ There was no evidence of an effect of maternal treatment in pregnancy on allergy-related outcomes at 9 years.^58^

### Nutrition

Through a collaboration led by colleagues at KEMRI-Wellcome Trust and the University of Oxford, extending the scope of our genetic studies, EMaBS was able to contribute to understanding the prevalence of nutritional deficiencies in the region, and links between nutritional deficiencies and infection. We contributed to estimates of the burden of iron deficiency,^59^ and its association with malaria^60^; and to evidence that the ferroportin Q248H mutation protects from anaemia, but not from malaria^61^; conversely, through Mendelian randomisation using sickle cell trait (which protects against malaria) as the instrumental variable, we contributed to findings that showed conclusively, for the first time, that malaria causes iron deficiency in African children.^62^ We also found that iron deficiency is associated with reduced malaria antibody levels, suggesting immunological consequences of this nutritional status.^63^

Similarly, EMaBS contributed to understanding the prevalence and risk factors for vitamin D deficiency in the region, with higher latitude, winter or rainy seasons and asymptomatic malaria among risk factors,^64^ and evidence of links between vitamin D and iron deficiency.^65^

Taking this collaboration forward, we plan to further explore the immunological implications of these nutrient deficiencies and their contribution to differences in vaccine responses such as those observed in the POPVAC trials.

### Cognition

It is widely believed that worm infections impair cognitive development and function, but analyses of such effects are often limited by confounding between worm infections and other factors important for child development.^66 67^ The EMaBS trials offered an opportunity to test causality through the randomised treatment of maternal worms in pregnancy and of soil-transmitted helminths in childhood. We adapted measures of development to our setting^68^ and tested 983 children aged 15 months in the EMaBS cohort. We found no effect of maternal treatment on childhood psychomotor or cognitive performance.^69^ A more detailed study among 163 EMaBS 5-year olds showed that being provided with a stimulating home environment was critical to cognitive development and could help to overcome the effects of other adverse exposures.^70^

Combining these assessments with the nutritional measurements described above showed that lower maternal and infant haemoglobin levels were associated with poorer psychomotor scores at 15 months but, reassuringly, neither of these was associated with impairment at 5 years.^71^ Similarly, we found that vitamin D status below 5 years was not associated with cognitive or motor outcomes at 5 years of age.^72^

### Cardiovascular disease risk

The concept of ‘Developmental Origins of Health and Disease’ (DOHaD) emphasises that health outcomes in adults may be determined early in life, including in utero.^73^ Much of the work that has been done in this field has taken place in relatively resource-rich settings, since few birth cohorts exist in tropical Africa. Prenatal and early-life exposures, including to infections, are different in this setting. We realised that the data collected in EMaBS, starting during the mother’s pregnancy, offered opportunities to explore DOHaD in the tropical African setting. When the children were 10 years old, we undertook a careful, cross-sectional, evaluation of cardiovascular disease risk factors, particularly blood pressure and lipid profiles, since these risk factors can track into later life. We found that rapid postnatal weight gain, particularly in low-birth-weight babies, was associated with increased blood pressure at 10 years of age, while there was little effect of birth weight per se.^74^ However, in a small substudy of body composition, fat mass index and free fat mass index were associated with birth weight, but not with postnatal growth rates.^75^ Family history, body mass index and waist circumference and current *Trichuris* infection were associated with higher blood pressure at 10 years, while malaria before age 5 years and fruit and vegetable consumption were associated with lower blood pressure.^76^ Current body mass index and current and prior malaria also showed associations with lipid profiles.^77^

### Research capacity strengthening

Alongside, and integral to, the research activities highlighted in the previous sections, the EMaBS cohort has provided extensive opportunities for strengthening the capacity of the research team and our collaborators. Major activities and achievements in this area include (1) supporting Entebbe Hospital staff to implement the first PMTCT programme at Entebbe Hospital, (2) training volunteers from the local area to become community field workers, who play a valuable role in shaping community engagement and enhancing participant follow-up, (3) ensuring continuing professional development of research team members, for example, through research skills training in good clinical research practice, good clinical laboratory research practice, human subjects protection, data protection and research project monitoring, and through clinical training such as in diagnosis of allergy-related diseases. We have been able to offer 75 internships to university students, and a total of 12 researchers have undertaken PhDs while others have completed diplomas, bachelors’ or masters’ degrees utilising and or building on data from EMaBS in their programmes.

## Future plans

EMaBS participants are now adults aged 19–22 years. Until recently, EMaBS core follow-up activities have included provision of healthcare for both participants and their mothers alongside research and data collection. As the participants make the transition to adulthood, we have adopted a new, less intensive model of contact for one-off study activities to address specific hypotheses, as exemplified by EMaBS@21. In a recent census exercise, we contacted and updated details of over 1500 participants, representing 64% of the original birth cohort, who all expressed willingness to take part in ongoing and future research initiatives.

Participant and stakeholder meetings held in 2024 included reflections from EMaBS participants, parents and the wider community on their role in, and impact of the study, and gathered information on which research topics and services would be of greater interest and benefit to the community for future activities. Particular areas highlighted included the role of genetics and environmental exposures in shaping risk of chronic diseases, the burden of mental health among young people and how it could be addressed, and the impact of climate change and urbanisation on health outcomes.

Building on our earlier findings, we are currently collecting comprehensive data on cardiovascular disease risk factors from EMaBS participants as they reach 21 years, in the ‘EMaBS@21’ project. The aim of EMaBS@21 is to understand how early-life exposures characteristic of tropical environments influence physiological determinants of cardiovascular diseases (CVDs) such as blood pressure, glucose, lipids, insulin sensitivity and BMI, measured in early adulthood, and hence risk of CVDs in later adulthood. The detailed protocol for this new round of data collection within the cohort has been published.^78^

Data and samples from EMaBS participants will also be central to the ‘Genetic causes and epigenetic consequences of infection in childhood (GECo)’ project led by colleagues at the University of Oxford. GECo will use data from EMaBS to investigate whether frequent childhood infections lead to specific epigenetic changes. This will be done by examining DNA methylation trajectories using stored peripheral blood mononuclear cells (PBMCs) from early childhood, 9 years, mid-teens and EMaBS@21, and relating these to existing data on early-life infection intensity. Later, the findings will be linked to those in cohort studies with older participants, such as MUL’s General Population Cohort,^79^ to determine whether epigenetic changes driven by infection exposure in EMaBS link to adverse outcomes in later life in the older cohorts.

## Strengths and limitations

There are relatively few longitudinal studies with detailed early-life and life-course data on nutrition, growth, infectious and sociodemographic exposures and long-term participant follow-up in low-income sub-Saharan African countries. To our knowledge, EMaBS is one of the longest-running and largest of such birth cohorts and thus represents a rare resource. Major strengths of the cohort include the extensive array of data on in utero, early childhood, adolescent and early adulthood exposures, which have been supplemented by additional data generated or planned through collaboration including genetic and epigenetic data and the availability of stored samples from multiple time points. These strengths are enhanced by strong participant and community engagement throughout the course of the study (and still ongoing), which has supported good participant retention.

Selection bias is a key potential limitation. This may have resulted initially from exclusion of pregnancies considered to be abnormal, although these were relatively few and previous work has suggested that cohort members have similar characteristics to others in the community.^80^ Cohort attrition was highest in the initial stages of the study, with 16% of live-born children not seen following birth. Excluding those who have died, over 50% of participants born into the cohort are still actively participating. However, we recognise the potential for bias induced by differences in characteristics of those who have, or have not been followed up. As a consequence of taking part in the study, cohort participants have received free medical care so their health outcomes may be better than in the general population. We feel the benefits of this provision outweigh the potential limitations as through engagement activities participants and their caregivers have consistently emphasised the importance of this provision in maintaining high follow-up rates. Our documentation of characteristics of mothers and children from the start, and the availability of comparative community data on some characteristics enables us to investigate these potential biases analytically. Characteristics of those retained are compared with those who are lost to follow-up. Regression analyses, used to control for confounding factors, can be expanded to incorporate inverse probability weights that capture the probability of inclusion for participants with specific characteristics.

The cohort sample size was designed to answer the primary research question relating to the impact of helminths and their treatment on responses to vaccines and incidence of infectious diseases. Therefore, the cohort is smaller than some birth cohorts, particularly those from high-income settings. Collaboration and data sharing with other research teams, as described below, are important for maximising resources and increasing statistical power. Finally, despite the extensive data collection, potential limitations in the use of EMaBS data for secondary analyses include the possibility of residual confounding from unmeasured confounders, particularly for new research questions that were not envisaged when the cohort was initiated. Sensitivity analyses can be used to investigate the degree of uncontrolled confounding that would substantially change conclusions.^81^

## Collaboration

EMaBS is a unique population cohort with an extensive collection of existing data and samples (table 1) from in utero to early adulthood, and further data and sample collection ongoing or planned: further information is available from the EMaBS website^16^ or from the corresponding author. Data and accompanying data dictionaries are available on reasonable request. Researchers interested in accessing the data can submit a formal request to the corresponding authors, detailing the data requested, the intended use for the data and evidence of relevant experience and other information to support the request. The request will be reviewed by the principal investigator in consultation with the MRC/UVRI and LSHTM Uganda Research Unit data management committee, with oversight from the UVRI and LSHTM ethics committees. Researchers given access to the data will sign data sharing agreements, which will restrict the use of data to the answering of prespecified research questions.

EMaBS has a strong track recording of partnering with other research teams, for example, through the Vaccgene consortium and as a member of the African Population Cohort Consortium and through sharing data for several systematic review and individual participant data meta-analyses.^82–84^ Our vision is to continue to work with EMaBS participants to address community-driven research questions, and to partner with other cohorts and researchers in Africa and beyond to address global health challenges.

## Data Availability

Data are available upon reasonable request.
