# Supplementary material for: Cohort profile: the Entebbe Mother and Baby Study (EMaBS)
Source: BMJ Open. 2026 Aug 26;16(8):e118325. doi: 10.1136/bmjopen-2026-118325 (PMC13536125; doi:10.1136/bmjopen-2026-118325)
Supplement: Supplementary file 1 [file bmjopen-2026-118325.reviewer_comments.pdf]

PEER REVIEW HISTORY

BMJ Open publishes all reviews undertaken for accepted manuscripts. Reviewers are asked to complete a checklist review form and are provided with free text boxes to elaborate on their assessment. These free text comments are reproduced below.

ARTICLE DETAILS

Title (Provisional)

Cohort profile: The Entebbe Mother and Baby Study (EMaBS)

Authors

Webb, Emily L; Walusimbi, Bridgious; Komata, Ronald; Elliott, Alison M; N/A, The EMaBS Study Team

---

VERSION 1 - REVIEW

**Reviewer**	**1**
**Name**	**Cooper, Philip**
**Affiliation**	**St George's University of London, Institute of Infection and Immunity**
**Date**	**11-May-2026**
**COI**	**None**

The manuscript clearly describes an extremely valuable and long-running African birth cohort, with major strengths in early-life infection data, vaccine/immunology outcomes, stored samples, genetics, nested trials, and current transition into adult cardiometabolic outcomes.

Comments:

1. Clarify attrition and representativeness - The manuscript notes 2,345 live births, 128 deaths and ~1,500 contactable participants from a recent census. It might be useful to more clearly show follow-up rates in the participant flow diagram to the present. Do the authors have any information from the recent census on how many are alive, contactable, and willing to participate (at latest follow-up).

2. Study limitations - The authors mention selection bias, attrition, free medical care, sample size and residual confounding. They should add how these limitations will be handled analytically in future work, especially inverse probability weighting, sensitivity analyses and comparison of retained versus lost participants.

3. Background information - There is a lot of information on study findings but relatively little in what is planned now. The authors could provide a Table or Figure showing more clearly the new study questions, and what they plan to measure and why?

4. Data access statement - It would be helpful to specify for data access if there is a formal application process, whether metadata dictionaries are available, etc.

5. Reference - {Zirimenya, 2025 #82}.

**Reviewer**	**2**
**Name**	**Al-Mekhlafi, Hesham**
**Affiliation**	**Jazan University, Medical Research Centre**
**Date**	**23-May-2026**
**COI**	**None**

This manuscript comprehensively describes nearly two decades of maternal and child health research in Uganda through a longitudinal cohort study, "The Entebbe Mother and Baby Study (EMaBS)". The manuscript presents the design and findings across different components of the study, including the primary hypothesis regarding the impact of helminth infections and deworming during pregnancy on childhood vaccine responses, as well as outcomes related to treatment safety during pregnancy, nutrition and cognition. It also highlights the study contributions to capacity building, research collaboration, and future research directions. Many of the study outcomes have been previously published elsewhere. Given the broad scope of the study and the diversity of methods employed, particularly the laboratory methods, it is understandably challenging to comprehensively include all aspects of the study within a single manuscript.

Overall, the manuscript is interesting, well-written and comprehensive in summarizing the cohort profile and its major outcomes. However, some specific comments provided below should be considered.

SPECIFIC COMMENTS

1. Title: The title is clear; however, it may be somewhat broad given the unique long-term focus and the wide range of research themes covered by the cohort.

2. Page 2, line 48: spell out PMTCT.

3. Page 2, STRENGTHS AND LIMITATIONS OF THIS STUDY: All listed points appear to describe strengths except point #4 which reflects a limitation. It should be clearly indicated as a limitation and can be moved to #5, after the strengths. In addition, other potential limitations of the cohort, such as selection bias indicated on page 15, can be added.

4. Page 3, line 42: The dose/regimen of albendazole used in the study can be specified.

5. Page 3, line 42: The “roundworms” can be changed to “soil-transmitted helminthes (STH)” or “intestinal nematodes”. Change “such as” to “including”.

6. Page 3, line 43: The regimen of albendazole for strongyloidiasis is different from other soil-transmitted helminthes including hookworm, Trichuris, and Ascaris.

7. Page 4, line 49: The dose/regimen of albendazole and praziquantel used in the study can be specified.

8. Page 7, line 8: Revise the citation “{Zirimenya, 2025 #82}”

9. Table 2: What about infections with other intestinal helminthes, including Trichuris, Ascaris and Strongyloides? Currently, only hookworm and Schistosoma mansoni infections are available in the table. Their inclusion would provide a more comprehensive overview of the cohort’s parasitic infection profile.

10. Table 2: Please state the time point represented by these results in the table caption (e.g. at the baseline or at enrollment) to improve clarity and interpretation of the results.

11. Table 2: Percent symbol (%) can be removed from all values in last column as it is indicated in the column header.

12. Page 12, line 53: change “soil-transmitted worms” to “soil-transmitted helminth”.

VERSION 1 - AUTHOR RESPONSE

COMMENTS FROM REVIEWER 1

Comment: The manuscript clearly describes an extremely valuable and long-running African birth cohort, with major strengths in early-life infection data, vaccine/immunology outcomes, stored samples, genetics, nested trials, and current transition into adult cardiometabolic outcomes.

Response*: We thank the reviewer for this positive comment.*

Comment: Clarify attrition and representativeness - The manuscript notes 2,345 live births, 128 deaths and ~1,500 contactable participants from a recent census. It might be useful to more clearly show follow-up rates in the participant flow diagram to the present. Do the authors have any information from the recent census on how many are alive, contactable, and willing to participate (at latest follow-up).

Response*: We have expanded the flow diagram (Figure 3) to show the updated figures for the census activity (which is still ongoing). We have also included additional information on the number of participants contactable in the text. There were 2,345 live births. To date, there have been 129 known deaths. From the ongoing census activity, 1,504 participants have been contacted so far and have confirmed their willingness to continue participating in cohort activities, while 623 could not be contacted. Participants who we have not yet attempted to contact are 89.*

Comment: Study limitations - The authors mention selection bias, attrition, free medical care, sample size and residual confounding. They should add how these limitations will be handled

analytically in future work, especially inverse probability weighting, sensitivity analyses and comparison of retained versus lost participants.

***Response**: Thank you. We have expanded this section to describe how the analytical approach in future work will handle these limitations. Specifically, we have explained how characteristics of retained participants will be compared to those of participants lost to follow-up, and that regression analyses used to control for confounders can be expanded to incorporate inverse probability weights that reflect characteristics of those retained versus those lost to follow-up, ensuring that models are representative of all original cohort members. We have also added text on pooling data with collaborators (to mitigate smaller sample size) and on how sensitivity analyses can be used to investigate the potential impact of residual confounding.*

Comment: Background information - There is a lot of information on study findings but relatively little in what is planned now. The authors could provide a Table or Figure showing more clearly the new study questions, and what they plan to measure and why?

Response: *Thank you for this suggestion. We are pleased to say that the protocol for the planned work (EMaBS@21) has now been published. We have included a sentence explaining this and provided the reference for the protocol paper:*

Sekitoleko et al. Early-life infectious and nutritional exposures and cardiovascular risk in early adulthood in Uganda: protocol for a new round of data collection in the Entebbe Mother and Baby Study birth cohort at 21 years (EMaBS@21). BMJ Open. 2026 Apr 7;16(4):e119342. doi: 10.1136/bmjopen-2026-119342.

Comment: Data access statement - It would be helpful to specify for data access if there is a formal application process, whether metadata dictionaries are available, etc.

***Response**: We have expanded the information under the Collaboration section to explain that data dictionaries would also be provided, and that the process described is the formal application process. Work is ongoing to include more detailed metadata on the EMaBS website, as has been done for the General Population Cohort at the MRC/UVRI and LSHTM Uganda Research Unit (https://www.lshtm.ac.uk/research/centres-projects-groups/general-population-cohort#gpc-resources which links to https://apps.mrcuganda.org/mrcdatavisibility/Home/GPC/). By directing researchers interested in accessing data to the EMaBS website our plan is that in the near future this will contain enhanced information on available data and samples.*

Comment: Reference - {Zirimenya, 2025 #82}.

***Response:** Thank you for flagging this. The reference has now been formatted correctly.*

COMMENTS FROM REVIEWER 2

Comment: This manuscript comprehensively describes nearly two decades of maternal and child health research in Uganda through a longitudinal cohort study, "The Entebbe Mother and Baby Study (EMaBS)". The manuscript presents the design and findings across different components of the study, including the primary hypothesis regarding the impact of helminth infections and

deworming during pregnancy on childhood vaccine responses, as well as outcomes related to treatment safety during pregnancy, nutrition and cognition. It also highlights the study contributions to capacity building, research collaboration, and future research directions. Many of the study outcomes have been previously published elsewhere. Given the broad scope of the study and the diversity of methods employed, particularly the laboratory methods, it is understandably challenging to comprehensively include all aspects of the study within a single manuscript.

Overall, the manuscript is interesting, well-written and comprehensive in summarizing the cohort profile and its major outcomes. However, some specific comments provided below should be considered.

***Response**: We thank the reviewer for their summary and positive comments.*

Comment: Title: The title is clear; however, it may be somewhat broad given the unique long-term focus and the wide range of research themes covered by the cohort.

***Response**: We thank the reviewer for their comment. The author guidelines for BMJ Open suggest this format for cohort profile papers and thus we have kept the title as is.*

Comment: Page 2, line 48: spell out PMTCT.

***Response**: We have now spelt out the acronym in full.*

Comment: Page 2, STRENGTHS AND LIMITATIONS OF THIS STUDY: All listed points appear to describe strengths except point #4 which reflects a limitation. It should be clearly indicated as a limitation and can be moved to #5, after the strengths. In addition, other potential limitations of the cohort, such as selection bias indicated on page 15, can be added.

***Response**: Thank you. We have now revised the strengths and limitations bullet points so that they are ordered with strengths first followed by limitations, and to indicate more clearly which are strengths and which are limitations. We have also included an additional bullet point relating to limitations as suggested.*

Comment: Page 3, line 42: The dose/regimen of albendazole used in the study can be specified.

***Response**: Thank you. We have included this information in the methods section on page 5, where we think it is best included. We have also specified the dosage for praziquantel.*

Comment: Page 3, line 42: The "roundworms" can be changed to "soil-transmitted helminthes (STH)" or "intestinal nematodes". Change "such as" to "including".

***Response**: We have made these changes as per the reviewer's suggestion.*

Comment: Page 3, line 43: The regimen of albendazole for strongyloidiasis is different from other soil-transmitted helminthes including hookworm, Trichuris, and Ascaris.

Response: We acknowledge this point. The trial was originally designed to investigate the impact of single-dose anthelminthic treatment as a simple intervention that would be straightforward to incorporate into routine antenatal care. Mothers found to have *Strongyloides* were treated with albendazole 400 mg daily for three days following delivery.

Comment: Page 4, line 49: The dose/regimen of albendazole and praziquantel used in the study can be specified.

Response*: Thank you – we have now incorporated this information.*

Comment: Page 7, line 8: Revise the citation "{Zirimenya, 2025 #82}"

Response*: Thank you for flagging this – we have corrected the format of this citation.*

Comment: Table 2: What about infections with other intestinal helminthes, including Trichuris, Ascaris and Strongyloides? Currently, only hookworm and Schistosoma mansoni infections are available in the table. Their inclusion would provide a more comprehensive overview of the cohort's parasitic infection profile.

Response*: Thank you – we have now included information on maternal Trichuris, Ascaris and Strongyloides in table 2. We have also included information on individual helminth species detected among the children at annual visits.*

Comment: Table 2: Please state the time point represented by these results in the table caption (e.g. at the baseline or at enrollment) to improve clarity and interpretation of the results.

Response*: The table 2 caption has been updated to clarify that these are characteristics of participants determined at birth and during early childhood, and characteristics of their mothers as determined at enrolment during pregnancy.*

Comment: Table 2: Percent symbol (%) can be removed from all values in last column as it is indicated in the column header.

Response*: Thank you – we have removed the percent symbols as suggested.*

Comment: Page 12, line 53: change "soil-transmitted worms" to "soil-transmitted helminth".

Response*: Thank you – we have made this change.*

VERSION 2 - REVIEW

**Reviewer**	**1**
**Name**	**Cooper, Philip**
**Affiliation**	**St George's University of London, Institute of Infection and Immunity**
**Date**	**19-Jun-2026**
**COI**	

The authors now cite a recently published protocol paper describing a new phase of follow-up within the Entebbe Mother and Baby Study cohort (Sekitoleko et al., 2026). This raises a question regarding the relationship between the current cohort profile and the previously

published protocol. It would be helpful for the authors to clarify how the objectives and content of the two papers differ, and specifically why both publications are needed. In particular, the manuscript would benefit from a brief explanation of the unique contribution of the present cohort profile beyond that provided in the protocol paper, and reassurance that there is no substantial overlap or duplication of previously published material.

**Reviewer**	**2**
**Name**	**Al-Mekhlafi, Hesham**
**Affiliation**	**Jazan University, Medical Research Centre**
**Date**	**22-Jun-2026**
**COI**	

The authors have adequately addressed all of my comments.

VERSION 2 - AUTHOR RESPONSE

COMMENTS FROM REVIEWER 1

Comment: The authors now cite a recently published protocol paper describing a new phase of follow-up within the Entebbe Mother and Baby Study cohort (Sekitoleko et al., 2026). This raises a question regarding the relationship between the current cohort profile and the previously published protocol. It would be helpful for the authors to clarify how the objectives and content of the two papers differ, and specifically why both publications are needed. In particular, the manuscript would benefit from a brief explanation of the unique contribution of the present cohort profile beyond that provided in the protocol paper, and reassurance that there is no substantial overlap or duplication of previously published material.

Response*: We thank the reviewer for raising this query. The current manuscript is a cohort profile paper and aims to (1) describe the original design, methodology and scope of the Entebbe Mother and Baby Study birth cohort, (2) summarise data collection procedures from the inception of the cohort in the early 2000s onwards, (3) describe key characteristics of cohort participants, (4) summarise the key findings to date, (5) provide insights into future plans, (6) discuss strengths and limitations, (7) summarise cohort resources and possibilities for potential collaboration. By contrast, the Sekitoleko paper is a protocol paper that describes the practicalities of a single cross-sectional round of data collection that is being done among EMaBS cohort members as they reach the age of 21 years, and analyses that are planned in relation to this data collection.*

The only small overlap between the two manuscripts is in the third paragraph of the "Future Plans" section of the current, cohort profile, manuscript. This section is a requirement of the "Cohort profile" format and in it we summarise, in four paragraphs, (1) the plans around the new less-intensive mode of participant contact, (2) possible future research directions informed by stakeholder meetings, (3) the aim and brief details of the EMaBS@21 work (the subject of the protocol paper) and (4) the aim and brief details of the GECO work (still undergoing ethical approvals).

We were pleased to be able to add the reference to the protocol paper in our response to the previous round of reviews, in order to be able to direct readers to where they could find out more

information on the EMaBS@21 data collection procedures. In order to further reduce this already minimal overlap, we have shortened the information provided about the EMaBS@21 work in the current manuscript (lines 437-443), and now just state its aim, and clarify that the Sekitoleko paper describes the protocol for this round of data collection.

We hope this reassures the reviewer that there is no substantial overlap or duplication of previously published material.

COMMENTS FROM REVIEWER 2

Comment: The authors have adequately addressed all of my comments.

Response*: We thank the reviewer for taking the time to re-review the paper, and for their helpful comments.*
